# Phylogenetic Analysis of Six-Domain Multi-Copper Blue Proteins

**DOI:** 10.1371/currents.tol.574bcb0f133fe52835911abc4e296141

**Published:** 2013-03-13

**Authors:** Andrey Vasin, Sergey Klotchenko, Ludmila Puchkova

**Affiliations:** Reseach Institute of Influenza; gomoResearch Institute of Influenza; St.-Petersburg State Polytechnical University

## Abstract

Multicopper blue proteins, composed of several repetitive copper-binding domains similar to one-domain cupredoxin-like proteins, were found in almost all organisms. They are classified into the three different groups, based on their two-, three- or six-domain organization. We found orthologs of chordate six-domain copper-binding proteins in animals, plants, bacteria and archea. The phylogenetic analysis of 183 multicopper blue proteins and their copper-binding sites comparison make us think that all the modern six-domain blue proteins have originated from the common ancestral six-domain protein in the process of gene duplication and copper-binding sites loss as a result of amino acid substitutions.

## Introduction

Multi-copper oxidases (MCOs) are an important class of enzymes catalyzing the four-electron transfer of molecular oxygen to water with concomitant one-electron oxidation of the substrate. MCOs include such proteins as laccases (E.C. 1.10.3.2), ascorbate oxidases (E.C. 1.10.3.3) and ferroxidases (E.C. 1.16.3.1) 1. Nitrite reductases (E.C. 1.7.2.1) also have structural similarity with MCOs, but they function as reductases **** 2. MCOs and nitrite reductases form the group of multi-copper blue proteins (MCBPs), consisting of one, two, three or six repetitive domains homologous to one-domain blue copper containing cupredoxins 1. Historically, copper atoms in proteins are classified into 3 types on the basis of their spectroscopic properties 3. Copper type 1 is often called "blue", because it is responsible for the characteristic blue color of cuproenzymes **** 2 ^,^ 4 ^,^ 5 ^,^ 6. The blue copper binding domains family includes a large number of phylogenetically distant homologues with sequence identity less than 10 %, while their structural organization by 8 β-sheets is conservative 7. MCBPs also contain interdomain copper types 2 and 3 binding sites. Amino acid residues that coordinate copper ions in blue copper proteins are highly conserved. The copper type 1 binding site is formed by two histidines, one cystein and one methionine 8. The first three amino acid residues play an essential role in copper ion coordination, while methionine can be changed by leucine or phenylalanine. Interdomain copper binding site in MCOs is trinuclear: 8 histidines (4 in each domain) coordinate one copper type 2 ion and two copper type 3 ions 8. For this reason MCBPs can be unambiguously identified on the basis of the relative location of copper coordinating amino acid residues in the protein primary structure.

The most diverse group in terms of amino acid sequences, functions and prevalence in organisms among all MCBPs is formed by three-domain MCOs, such as laccases, ascorbate oxidases and yeast ferroxidases Fet3 9. They were found in bacteria, fungi, insects and plants 10 ^,^ 11. Six-domain MCBPs, such as ceruloplasmin (Cp), hephaestin (Heph) and blood coagulation factors (BCFs) V and VIII, have been found only in vertebrates until recently. The most studied six-domain MCBP is Cp – a polyfunctional multicopper ferroxidase (MCFO), an important component of copper metabolic system and the central participant of iron metabolism 12 ^,^ 13. Intracellular Cp isoforms are universal copper transporters along intercellular communication pathways, while membrane-associated Cp isoforms act as ferroxidases, participating in the bidirectional iron ions transport through the cellular membranes. Heph, another MCFO, is located on the apical surface of enterocytes, where it catalyzes the oxidation of ferro- to ferri-ion, enabling safe iron absorption from the gastrointestinal tract. All six-domain MCBPs consist of six tandem homologous domains; the exceptions are factors V and VIII, which contain two additional regions 14 ^,^ 15 ^,^ 16. Cp and Heph have three mononuclear blue copper binding sites in domains 2, 4, 6 and one trinuclear copper coordinating center between domains 1 and 6 (Fig. 1). Factor VIII contains one trinuclear and two mononuclear copper-binding sites in domains 2 and 6. Factor V is the only MCBP, that does not have any conservative amino acid residues, coordinating copper ions.

**The general scheme of six-domain MCBPs evolution.**
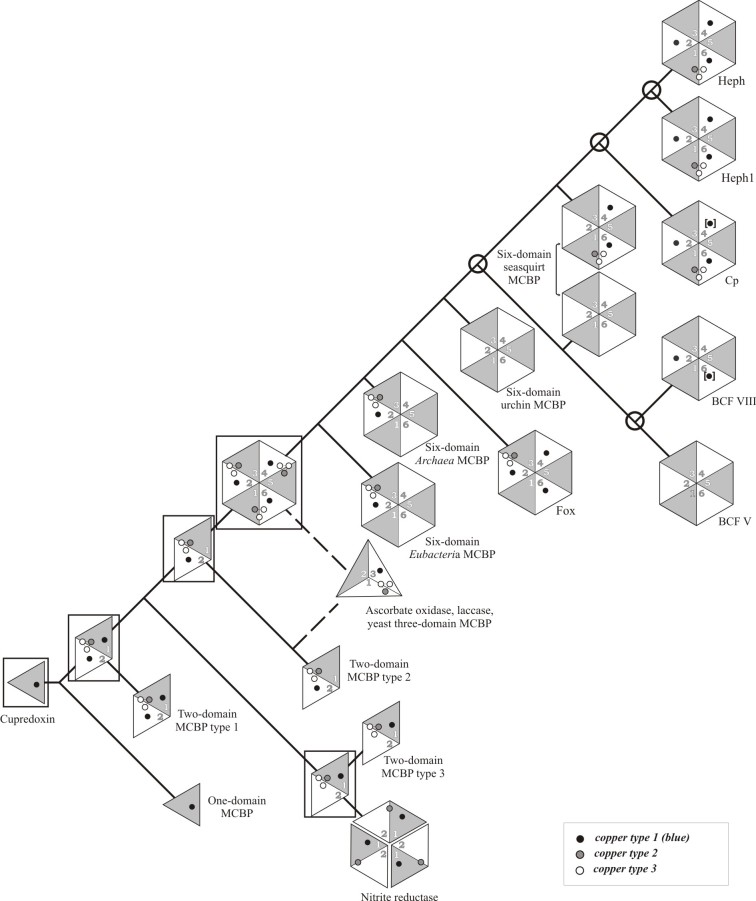
The duplication of the parental cupredoxin led to the formation of three types of two-domain proteins with trinuclear copper binding site. The triplication of one of them led to the formation of a hypothetical six-domain protein, from which all modern six- and, possibly, three-domain MCBPs were desended. Hypothetical evolutional ancestors are framed. Two possible evolutional pathways of three-domain proteins formation are marked with dotted lines. Gene duplication events that led to the appearance of five homologous MCBPs in mammals are marked with bubbles. Copper binding sites that could be absent in the proteins of several biological species are indicated in square brackets. MCBP – multicopper blue protein; Cp – ceruloplasmin; BCF – blood coagulation factor; Fox – algae multicopper ferroxidase; Heph – hephaestin; Heph1 – hephestin-like protein 1.

In spite of the significant phylogenetic divergence and even different functions of MCBPs, the observed similarity of their domain organization and amino acid sequences indicate their common ancestry (Fig. 1). However many aspects of MCBPs evolution remain unknown. In this paper we performed the phylogenetic analysis of six-domain MCBPs and supplemented the existing schemes of their origin and evolution.

## Materials and methods

Amino acid sequences were obtained from the GenPept protein sequences database (http://www.ncbi.nlm.nih.gov/protein/) using the BLASTP program 17. Cp (NP_036664.1) and MnxG (ZP_02951893.1) sequences were used as queries. Sequences with E-value < 0.001 were chosen as significantly similar. In order to exclude we one-, two- and three-domain MCBPs we selected only mononuclear and/or trinuclear copper binding sites containing sequences over 800 amino acids long. Multiple alignment of amino acid sequences was made using Seaview software 18 using MUSCLE algorithm **** 19. Alignments of prokaryotic and eukaryotic MCBP were overlapped manually using BioEdit software 20 on the basis of the conservative copper binding sites positions. The obtained alignments were used for phylogenetic analysis in MEGA4 21 using neighbor-joining method, phylogenetic distances were evaluated using Poisson correction model 22. Positions with alignment gaps were eliminated for pairwise alignments. Topology reliability was checked using bootstrap analysis on the basis of 1000 replicates. Protein sequences were designated in accordance with GenPept accession numbers. Organisms were classified in accordance with NCBI taxonomic hierarchy (http://www.ncbi.nlm.nih.gov/Taxonomy).

## Results

The phylogenetic tree for eukaryotic six-domain MCBPs (see appendix 1) is presented on Fig. 2. The majority of the sequences are categorized into two groups consisting of MCFOs and BCFs V and VIII. The MCFOs cluster consists of Cp and Heph subclusters, similarly BCFs cluster consists of factors V and VIII subclusters. Cp subcluster is clearly divided into 3 groups in concordance with the taxonomic classification: mammals, birds and fish. Since Cp coding gene has just one copy in the genomes of vertebrates 23 ^,^ 24 ^,^ 25, species-specific Cp protein isoforms are grouped in individual clades. Heph subcluster consists of two groups, corresponding to Heph and Heph-like protein 1 (Heph1), encoded by two different genes. Just like for Cp there is a reliable division between mammalian and birds proteins inside of each of these two groups. The only sequence of fish Heph ortholog (CAG00485.1) is located inside Heph sub-cluster on the separate branch of the phylogenetic tree. This fact suggests that the duplication of a Heph precursor gene could occur already after the superclass of terrestrial vertebrates had appeared. Both clusters described above share the closest common ancestor with two lancelet *Branchiostoma floridae *sequences, XP_002593056.1 and XP_ 002593057.1, which are encoded by two genes with 55% identity. It is possible, that each of these sequences has a common ancestry with Cp and Heph, correspondingly.

**Neighbor-joining phylogenetic tree for 157 eukaryotic six-domain multicopper blue proteins.**
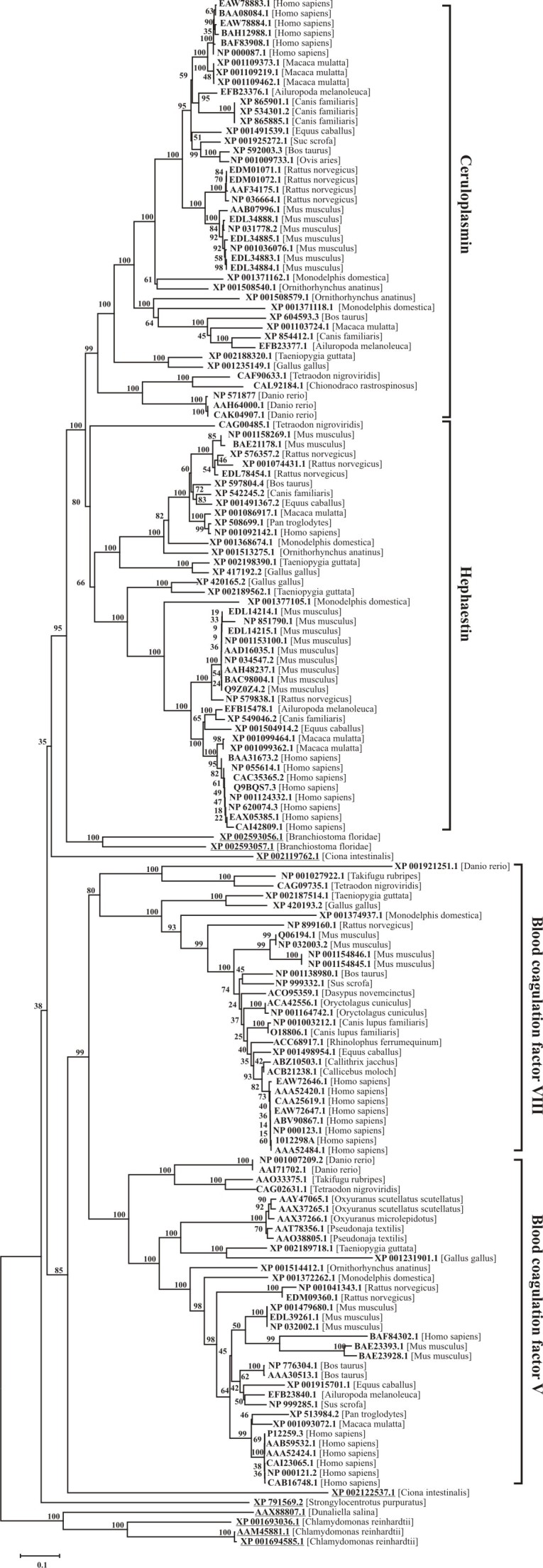
Evolutional distances were estimated as the number of amino acids substitutions per site, considering Poisson correction. The bootstrap values were evaluated on the base of 1000 pseudoreplicates. The list of protein sequnces is presented in Appendix 1.

The cluster of BCFs is also split into subclusters of factors V and VIII sequences, which are further categorized in a class-specific manner into 3 groups, corresponding to mammals, birds and fish. Amino acid sequences of oscutarin and pseutarin, two factor V - similar snake prothrombin activator components, are related to the group composed of bird’s factor V proteins sequences 26 ^,^ 27. Amino acid sequence divergence within the BCFs is greater than within MCFOs cluster. BCFs variability could be explained by the complete or partial lack of oxidative function as a result of the disturbance of the copper-binding sites structure and the different mechanisms of BCFs processing.

The revealed BCFs form a common clade with sequence XP_002122537.1 from sea squirt *Сiona* *intestinalis*. In turn MCFOs share a common ancestor with another *Сiona* *intestinalis *sequence XP_002119762.1, however the reliability of the appropriate node is small. All protein sequences mentioned above are related to chordates and form a common clade with sea urchin *Strongylocentrotus purpuratus’s* sequence XP_791569.2.

Until recently six-domain MCBPs were considered to relate to animal world only. However MCBPs Fox1 and Fox2 were found in green algae* Chlamydomonas reinhardtii *and Fox1 homologue was found in *Dunaliella salina* 28. According to our analysis these unicellular algae MCFOs form a phylogenetically distinct cluster. Since the phylogenetic divergence between unicellular algae and animals, this cluster could be accepted as an outer group for all to date known animal six-domain MCBPs.

The existance of six-domain MCBPs in unicellular algae made us think that six-domain MCBPs could be found in bacteria. Earlier it was reported that the manganese oxidase MnxG, remotely similar to MCBPs 29, was found in bacterium *Clostridium perfringens.* We also revealed MnxG sequence (ZP_02951893.1) by BLASTP analysis, but since the low degree of sequence identity between MnxG and the majority of eukaryotic MCBPs the expectation value was not significant (E-value = 2.5). For this reason an additional BLASTP analysis using MnxG as a query was made in order to find its homologues. We revealed 23 amino acid sequences from 1217 to 2681amino acids long with the significant degree of the identity to MnxG (E-value < 0,001) (see Appendix 2). Most sequences belong to *Eubacteria* with the exception of two *Archaea* proteins from *Halorubrum lacusprofundi *and *Haloterrigena turkmenica*. Fox1 sequence (XP_001694585.1) has been used for the multiple protein alignment construction as a phylogenetically remote six-domain MCBP with a known structure. The phylogenetic tree constructed on the basis of this alignment is presented on Fig. 3. All sequences could be divided into two clusters. The first cluster contains MCO-like protein sequences from *Proteobacteria*. The second one consists of MnxG-like bacterial proteins from *Bacillus* and *Clostridium *genera (*Firmicutes* phyla) and two sequences that belong to *Halobacteria*. Both clusters have a common node with ZP_01851853.1 sequence, which belongs to *Planctomycetes.*The obtained data is not sufficient to make any valid conclusions, however we can speculate that there could be a common parental sequence for these MCO-like proteins in some progenitor prokaryotic organisms.

**Neighbor-joining phylogenetic tree for 23 putative prokaryotic six-domain multicopper blue proteins, homologous to Mn-oxidase:**
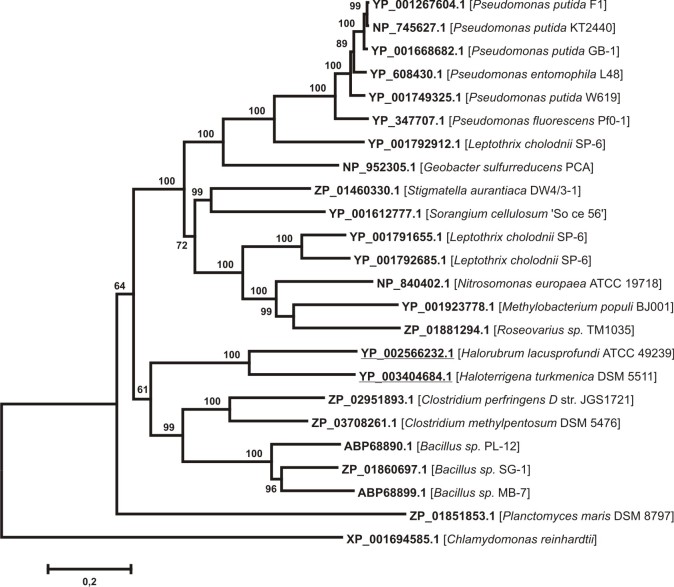
Evolutional distances were estimated as the number of amino acids substitutions per site, considering Poisson correction. The* Archea* sequences are underlined. XP_001694585.1 protein sequence from *Chlamydomonas reinhardtii* was used as outgroup. The list of protein sequences is presented in Appendix 2

Reconstruction of the possible six-domain MCBPs evolution pathways requires the comparison of MCBPs sequences from the phylogenetically remote species. However, since the low degree of the total sequence identity between MCBPs from eukaryotic and prokaryotic species we further analyzed the conservative amino acid composition of copper-binding domains, which define the structure and functions of MCBPs. For this purpose we manually matched the multiple sequence alignments of eukaryotic MCBPs and their bacterial homologues according to the copper-binding domains coordinates along Fox1 sequence (XP_001694585.1), presented in both alignments. Further we selected 19 most typical MCBPs representatives, containing all the found types of copper-binding sites composition (see Fig. 4). It can be seen, that there is at least one copper- binding site in the majority of the represented sequences. The exceptions are sequences XP_791569.2 from *Strongylocentrotus purpuratus*, XP_002122537.1 from *Сiona intestinalis*and factor V of vertebrates. In factor V the amino acid residues, that form copper-binding sites, have been partially conserved. The fact, that there is a common node between XP_002122537.1 and BCFs V and VIII sequences in the phylogenetic tree, can indicate the existance of their common ancestor.

**Amino acid sequence alignment of copper-binding centers of MCBPs from remote phylogenetic lines.**
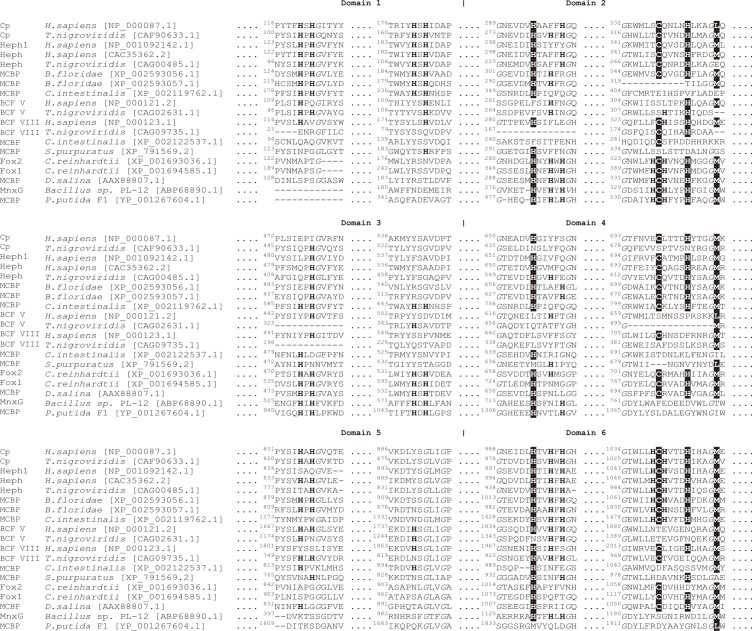
Amino acids, forming the copper-binding mononuclear centers, are marked with black background. Amino acids, forming the copper-binding trinuclear centers, are marked with a bold font. Conservative glycine amino acids residues are marked with italic font. The GenPept sequence numbers are indicated in square brackets. Cp – ceruloplasmin; Heph – hephaestin, Heph1 – hefaestin-like protein; MCBP – multicopper blue protein; factor V – blood coagulation factor V; factor VIII – blood coagulation factor VIII; Fox1 и Fox2 – multicopper ferroxidase from *Chlamydomonas reinhardtii*; MnxG – Mn-oxidase.

The most conservative mononuclear copper-binding sites are located in domains 2 (absent in sequences XP_002593057.1 from *Branchiostoma floridae*, XP_002119762.1 from *Сiona* *intestinalis*, factor VIII from *Tetraodon nigroviridis* and XP_001693036.1 from *Chlamydomonas reinhardti*) and 6 (absent in prokaryotic proteins) of six-domain MCBPs. Mononuclear copper-binding site, located in domain 4, was found only in Fox, Heph and Cp (except fish Cp) sequences.

So far, it was considered that the only trinuclear copper-binding site is located between domains 1 and 6 of six-domain MCBPs , however, as it appeared, its location could be different. Histidine amino acid residues forming trinuclear copper-binding site in MCFOs from *Chlamydomonas reinhardtii *and* Dunaliella salina *are located in domains 2 and 3. The same localization of interdomain copper-binding site was found in all the analyzed bacterial six-domain MCOs; moreover, their mononuclear sites are localized just in domain 2. It is interesting, that in the analyzed sequences there are some residual amino acids from mononuclear as well as trinuclear copper-binding sites, that were localized between domains 1 and 6, 2 and 3, and in a less degree between 4 and 5, and possibly have been eliminated during evolution.

## Discussion

One-domain cupredoxin is presumably the evolutional precursor of blue copper binding domains, whose multiplication and subsequent modification led to the formation of multi-domain MCBPs with typical mono- and trinuclear copper-binding sites 30. The general scheme of the MCBPs evolution, proposed by *Ryden and Hunt* 31 and *Murphy et al* 5, implies the presence of the tentative ancestral six-domain protein containing 3 trinuclear and 3 mononuclear copper-binding sites (Fig. 1.). Six-domain MCBPs are typical for vertebrates, but the existence of their homologues in green algae and some archea and bacterial species makes us think about their prokaryotic origin. In spite of the sparse distribution of six-domain MCBPs in prokaryotes there are some arguments for their common ancestry with eukaryotic MCBPs, but not for sporadic horizontal transfer. First, MCBPs were found in almost all known eukaryotic as well as prokaryotic organisms. Second, the main functions of MCBPs are associated with the presence of copper-binding sites, whose structure is very conservative among all known MCBPs (see Fig. 4). Third, the domain structure of MCBPs, as a rule, is strictly species-specific. In eukaryotes only three- (yeast and **higher plants) or six-domain (animals and green algae) MCBPs were found. Most of the prokaryotic genomes encode one- and two-domain MCBPs, however there are some exceptions. In particular, 17 of 23 prokaryotic genomes regarded here encode only six-domain MCBPs, while the remaining 6 genomes, including two archea species, encode additional two-domain MCBPs.

Despite of the significant phylogenetic divergence between MCBPs their general structure, defined by the copper-binding sites, as a whole remains unchanged. Blue copper-binding sites in the hypothetical ancestral six-domain MCBPs were expected to be localized in domains 2, 4 and 6 and inter-domain trinuclear copper binding sites – between domains 1 and 6, 2 and 3, 4 and 5. Probably further during the evolution these sites were partly or even completely lost. In the contemporary six-domain MCBPs the number of mononuclear sites varies from 0 to 3 and the number of trinuclear sites does not exceed 1. Moreover trinuclear copper binding sites were only found between domains 1 and 6, like in MCBPs of chordates, **or between domains 2 and 3, like in MCBPs of green algae, some bacteria and archea species. No MCBPs with trinuclear center located between domains 4 and 5 were described so far. However, multiple protein alignment of MCBPs copper-binding sites showed that the copper coordinating amino acid residues are partially remained for some lost mono- as well as tri-nuclear copper-binding sites in all the analyzed proteins.

The chain of events of the modifications of the hypothetical ancestral six-domain MCBPs may be as follows. The loss of two trinuclear copper-binding sites, located between domains 1 and 6 and domains 4 and 5, has led to the occurrence of green algae MCBPs 28 and prokaryotic MCBPs, which also lost two of the three blue copper binding sites in domains 4 and 6 29 ^,^ 32. In turn, the loss of two trinuclear sites, located between domains 2 and 3 and domains 4 and 5, resulted in the formation of eukaryotic six-domain MCBPs, like Cp, Heph, BCFs V and VIII. Two potential MCBPs from *Tetraodon nigroviridis* and *Chionodraco rastrospinosus* have the same structure except for the missing blue copper-binding site in domain 4.

There were at least four gene duplication events (see Fig. 1) in higher eukaryotic organisms that resulted in the appearance of at least five homologous MCBPs in mammals. The first duplication of the ancestral six-domain MCBP gene probably took place not later, then on the early stages of chordate’s evolution, since two different MCBPs were found in sea squirts, while sea urchin’s MCBP gene is presented by a single copy in the genome. This duplication led to the formation of two groups of MCBPs with completely different functions: MCFOs and BCFs V and VIII. The subsequent independent duplications of these genes resulted to the appearance of BCFs V and VIII genes and Cp and Heph genes, found in vertebrates. The time of these duplications cannot be estimated on the base of the available data, although lancelet *Branchiostoma floridae* has two MCFO-like proteins. Finally Heph gene duplication probably took place after the sub-class of terrestrial animals had appeared. The general scheme of the modern six-domain MCBPs formation from the hypothetical six-domain ancestral protein is presented on Fig. 1.

## Conclusion

The phylogenetic analysis of six-domain MCBPs allows to assume that they have a common prokaryotic six-domain protein ancestor and were formed through the chain of gene duplications and amino acids substitutions which resulted in the loss of a part of copper-binding sites.

## Appendix 1

### Six-domain multicopper blue proteins, used for the phylogenetic analysis

**Table:**

GenBank acc. no.	Organism	GenBank name	Length, а.a.
EFB15478. 1EFB23376.1 EFB23377.1 EFB23840.1	*Ailuropoda melanoleuca*	PANDA_018040* PANDA_016788* PANDA_016789* PANDA_007108*	1158 1060 909 2211
AAA30513.1 NP_001138980.1 NP_776304.1 XP_592003.3 XP_597804.4 XP_604593.3	*Bos taurus*	Factor V Factor VIII Factor V ceruloplasmin* hephaestin* ceruloplasmin*	2206 2323 2211 1060 1157 1055
XP_002593056.1 XP_002593057.1	*Branchiostoma floridae*	BRAFLDRAFT_74383* BRAFLDRAFT_74384*	1194 1057
ACB21238.1	*Callicebus moloch*	Factor VIII	1167
ABZ10503.1	*Callithrix jacchus*	Factor VIII	1892
NP_001003212.1 O18806.1 XP_534301.2 XP_542245.2 XP_549046.2 XP_854412.1 XP_865885.1 XP_865901.1	*Canis familiaris*	Factor VIII Factor VIII Ceruloplasmin* hephaestin* hephaestin* ceruloplasmin* ceruloplasmin* ceruloplasmin*	2343 2343 1065 1160 1198 1107 1073 1094
CAL92184.1	*Chionodraco rastrospinosus*	ceruloplasmin	1069
AAM45881.1 XP_001693036.1 XP_001694585.1	*Chlamydomonas reinhardtii*	Cu-ferroxidase Cu-oxidase Cu-ferroxidase	1142 1089 1142
XP_002119762.1 XP_002122537.1	*Ciona intestinalis*	hephaestin* pseutarin C*	16701434
AAH64000.1 AAI71702.1 CAK04907.1 NP_001007209.2 NP_571877.1 XP_001921251.1	*Danio rerio*	ceruloplasmin Factor V ceruloplasmin Factor V ceruloplasmin Factor VIII*	1087 2087 1100 2101 1087 852
ACO95359.1	*Dasypus novemcinctus*	Factor VIII*	2295
AAX88807.1	*Dunaliella salina*	ferroxidase	1075
XP_001491367.2 XP_001491539.1 XP_001498954.1 XP_001504914.2 XP_001915701.1	*Equus caballus* * *	hephaestin* ceruloplasmin* Factor VIII* hephaestin* Factor V*	1275 1068 2331 1161 2107
AAO33367.1♦ XP_001231901.1 XP_001235149.1 XP_417192.2 XP_420165.2 XP_420193.2	*Gallus gallus*	Factor VIII Factor V* ceruloplasmin* hephaestin* hephaestin* Factor VIII*	1377 1880 1079 1151 1112 1709
1012298 AAAA52420.1 AAA52424.1 AAA52484.1 AAB59532.1 ABV90867.1 BAA08084.1 BAA31673.2 BAF83908.1 BAF84302.1 BAF85591.1♦ BAH12988.1 CAA25619.1 CAB16748.1 CAC35365.2 CAI23065.1 CAI42809.1 EAW72646.1 EAW72647.1 EAW78883.1 EAW78884.1 EAX05385.1 NP_000087.1 NP_000121.2 NP_000123.1 NP_001092142.1 NP_001124332.1 NP_055614.1 NP_620074.3 P12259.3 Q9BQS7.3	*Homo sapiens* * *	Factor VIII Factor VIII Factor V Factor VIII Factor V Factor VIII ceruloplasmin* KIAA0698 no name* no name* no name* no name* no name* Factor V hephaestin Factor V hephaestin Factor VIII Factor VIII ceruloplasmin ceruloplasmin hephaestin ceruloplasmin Factor V Factor VIII hephaestin hephaestin hephaestin hephaestin Factor V hephaestin	2351 2351 2224 2351 2224 1457 1006 1104 1065 1030 1090 946 2351 2224 1158 2229 811 2096 2351 1072 1018 1161 1065 2224 2351 1159 1160 891 1212 2224 1158
XP_001086917.1 XP_001093072.1 XP_001099362.1 XP_001099464.1 XP_001103724.1 XP_001109219.1 XP_001109373.1 XP_001109462.1	*Macaca mulatta*	hephaestin* Factor V* hephaestin* hephaestin* ceruloplasmin* ceruloplasmin* ceruloplasmin* ceruloplasmin*	1202 2190 891 1158 999 1072 1069 1051
XP_001368674.1 XP_001371118.1 XP_001371162.1 XP_001372262.1 XP_001374937.1 XP_001377105.1	*Monodelphis domestica*	hCG1810904* ceruloplasmin* ceruloplasmin* Factor V* Factor VIII* hephaestin*	1076 1073 1060 2182 960 1226
AAB07996.1 AAD16035.1 AAH48237.1 BAC98004.1 BAE21178.1 BAE23393.1 BAE23928.1 EDL14214.1 EDL14215.1 EDL34883.1 EDL34884.1 EDL34885.1 EDL34888.1 EDL39261.1 NP_001036076.1 NP_001153100.1 NP_001154845.1 NP_001154846.1 NP_001158269.1 NP_031778.2 NP_032002.1 NP_032003.2 NP_034547.2 NP_851790.1 Q06194.1 Q9Z0Z4.2 XP_001479680.1	*Mus musculus* * *	ceruloplasmin hephaestin hephaestin mKIAA0698 no name* no name* no name* hephaestin hephaestin ceruloplasmin ceruloplasmin ceruloplasmin ceruloplasmin Factor V ceruloplasmin hephaestin Factor VIII Factor VIII hephaestin ceruloplasmin Factor V Factor VIII hephaestin hephaestin Factor VIII hephaestin Factor V*	1062 1157 1156 1169 911 1016 1012 1169 1168 1085 1059 1108 1060 2136 1086 1156 2249 2247 1159 1061 2183 2319 1157 847 2319 1157 2157
XP_001508540.1 XP_001508579.1 XP_001513275.1 XP_001514412.1	*Ornithorhynchus anatinus*	ceruloplasmin* ceruloplasmin* no name* Factor V*	962 980 1390 1990
ACA42556.1 NP_001164742.1	*Oryctolagus cuniculus*	Factor VIII Factor VIII	2222 2336
NP_001009733.1	*Ovis aries*	ceruloplasmin	1048
AAX37266.1	*Oxyuranus microlepidotus*	pseutarinC	1460
AAX37265.1 AAY47065.1	*Oxyuranus scutellatus*	oscutarin C oscutarin С	1459 1458
XP_508699.1 XP_513984.2	*Pan troglodytes*	no name* Factor V*	1298 1812
AAO38805.1 AAT78356.1	*Pseudonaja textilis*	pseutarin C Factor V	1460 1459
AAF34175.1 EDL78454.1 EDM01071.1 EDM01072.1 EDM09360.1♦ NP_001041343.1 NP_036664.1 NP_579838.1 NP_899160.1 XP_001074431.1 XP_576357.2	*Rattus norvegicus*	ceruloplasmin hephaestin* ceruloplasmin ceruloplasmin Factor V Factor V ceruloplasmin hephaestin Factor VIII hephaestin* hephaestin*	1084 914 1059 1084 1661 2102 1059 1157 2258 875 1180
ACC68917.1	*Rhinolophus ferrumequinum*	Factor VIII*	2352
XP_791569.2	*Strongylocentrotus purpuratus*	ferroxidase*	1047
NP_999285.1 NP_999332.1 XP_001925272.1	*Sus scrofa*	Factor V Factor VIII ceruloplasmin*	2258 2133 1065
XP_002187514.1 XP_002188320.1 XP_002189562.1 XP_002189718.1 XP_002198390.1	*Taeniopygia guttata*	Factor VIII* ceruloplasmin* hephaestin* Factor V* hephaestin*	1473 1057 1200 1936 1074
AAO33375.1 NP_001027922.1	*Takifugu rubripes*	Factor V Factor VIII	1802 1639
CAF90633.1 CAG00485.1 CAG02631.1 CAG09735.1	*Tetraodon nigroviridis*	no name no name no name no name	1048 1007 1725 1304

Comments. Names of proteins are given according to their annotations in GenPept. *No name* – the protein does not have a name in GenBank. * - protein sequence is predicted by computer based annotation.^ ♦^- sequence was not used for the construction of phylogenetic tree.

## Appendix 2

### Prokaryotic six-domain multicopper blue proteins, used for the phylogenetic analysis

**Table:**

GenBank acc. no.	Organism	GenBank name	Length, а.a.
ABP68899.1 ABP68890.1 ZP_01860697.1	*Bacillus sp. *MB-7 *Bacillus sp. *PL-12 *Bacillus sp*.SG-1**	MnxG MnxG BSG1_00155*	1223 1227 1217
ZP_03708261.1 ZP_02951893.1	*Clostridium methylpentosum *DSM 5476 *Clostridium perfringens D *str.**JGS 1721**	CLOSTMETH03020* MnxG	1231 1297
NP_952305.1	*Geobacter sulfurreducens *PCA**	GSU1252*	1652
YP_002566232.1	*Halorubrum lacusprofundi *ATCC 49239**	Hlac_1576*	1525
YP_003404684.1	*Haloterrigena turkmenica *DSM 5511**	Htur_3146*	1476
YP_001791655.1 YP_001792685.1 YP_001792912.1	*Leptothrix cholodnii *SP-6 *Leptothrix cholodnii SP-6Leptothrix cholodnii SP-6* **	MCO* MCO* Lcho_3893*	1569 1581 2016
YP_001923778.1	*Methylobacterium populi *BJ001**	MCO*	2493
NP_840402.1	*Nitrosomonas europaea *ATCC 19718**	MCO	1886
ZP_01851853.1	*Planctomyces maris *DSM 8797**	МCО*	2047
YP_608430.1 YP_347707.1 YP_001267604.1 YP_001668682.1 NP_745627.1 YP_001749325.1	*Pseudomonas entomophila *L48 *Pseudomonas fluorescens *Pf0-1 *Pseudomonas putida *F1 *Pseudomonas putida *GB-1 *Pseudomonas putida *KT2440 *Pseudomonas putida *W619**	PSEEN2857* Pfl01_1975* Pput_2281* PputGB1_2447* PP_3490* PputW619_2456*	1947 1941 1945 1944 1970 1959
ZP_01881294.1	*Roseovarius sp. *TM1035**	MCO*	2681
YP_001612777.1	*Sorangium cellulosum *'So ce 56'**	MCO*	1580
ZP_01460330.1	*Stigmatella aurantiaca *DW4/3-1**	MnxG*	1760

Comments. Names of proteins are given according to their annotations in GenPept. * - sequence of a protein is predicted with computer based annotation.
